# New Drugs and Preparations

**Published:** 1894-05-05

**Authors:** 


### NEW DRUGS AND PREPARATIONS.

[We shall be glad to receive, at our Office, 428, Strand, London, W.C., from the manufacturers, specimens of all new preparations and drugs which may be brought out from time to time.]

### GELSEMIUM SEMPERVIRENS.

This powerful drug, which has been much neglected of late years, has recently been subjected to a thorough examination by Cushony,[1] who gives references to the more important literature of the subject. He points out that there exist the most discordant statements as to the action of gelsemium, but that this is probably due to the fact that different preparations of it contain different strengths of the active alkaloids. Of these there are two—viz., gelsemin first isolated by Gerrard, and gelseminum isolated by Thompson.

Gelsemin is a white dry amorphous powder with a strong alkaline reaction, insoluble in water, soluble in ether; it forms salts, of which the chloride and sulphate are crystalline, fairly soluble in water, and precipitated by alcohol ether.

Gelseminin is an amorphous resinous substance of a yellowish-brown colour, slightly soluble in water, and very soluble in alcohol and ether. Its salts are all amorphous, and are much more soluble in water than those of gelsemin.

*Physiological Action of Gelsemin.*—This alkaloid has a different action on the frog, according to the species employed; in some a narcotic action is observed, in others there is an increase in the reflex excitability, closely resembling that seen after strychnine is given. It is not a very active poison, for in frogs the minimal dose necessary to produce toxic symptoms is 5 milligrammes. It kills by interference with respiration; and in the species in which the reflex excitability of the cord is at first increased, ultimately it is depressed, and at the same time the end plates of the motor nerves are paralysed. Gelsemin is said to cause, when dropped into the eye, temporary ciliary injection and contraction of the pupil, soon followed by mydriasis. There was no action on the accommodation, but it is almost certain that these effects are due to an admixture of gelseminin, and that gelsemin has no mydriatic action.

*Action of Gelseminin.*—This alkaloid is much more powerful than gelsemin. One milligramme in frogs causes slight narcosis, together with a curious transitory tremor of the head and shoulders. Larger doses cause paralysis of the end plates of the motor nerves, and the action of this alkaloid on frogs may be summed up by saying that firstly there is a paralysis of the central nervous system descending from the brain to the spinal cord, and secondly a paralysis of the endings of the motor nerves and of the vagus. The tremors seem to be central. Gelseminin is so very powerful on mammals in comparison with gelsemin, that all the poisonous symptoms induced in man by the gelsemium sempervirens are due to the gelseminin in it. In mammals it has a powerful action on the respiratory centre, acting as a depressant, but its curara-like action on the ends of the motor nerves is absent probably because the depression of the respiratory centre kills before there is time for it to be developed. Gelseminin has no action on the circulation, and probably it does not cause a lowering of the bodily temperature, although some authors maintain that it does. It has a powerful local action on the eye. The first thing noticed is reddening and smarting of the conjunctiva, but this soon passes off, the pupil then begins to dilate and soon after the power of accommodation is lost. The whole action is very slow, and it is sometimes three days before the effect of a single application has passed off. We may conclude that gelsemium sempervirens is of no use as a therapeutic agent. At one time it was thought that possibly it might be useful for ophthalmic surgery, but it is too irritating, and its action is too slow for it to be of any practical value as a mydriatic.

[1] Practitioner, July, 1893.

